# Seasonality of acute kidney injury incidence in Japanese outpatients

**DOI:** 10.1038/s41598-026-61190-6

**Published:** 2026-07-27

**Authors:** Yuka Sakazaki, Yuki Kondo, Mizuki Okuma, Ayaka Seki, Takamasa Sakai, Tetsumi Irie, Yoichi Ishitsuka

**Affiliations:** 1 Department of Clinical Chemistry and Informatics, Graduate School of Pharmaceutical Sciences, Kumamoto University, 5-1 Oehommachi Chuo-ku, Kumamoto, 862-0973 Japan; 2 Drug Informatics, Faculty of Pharmacy, Meijo University, 150 Yagotoyama, Tempaku-ku, Nagoya, Japan; 3 Department of Pharmaceutical Packaging Technology, Faculty of Life Sciences, Kumamoto University, 5-1 Oehommachi Chuo-ku, Kumamoto, Japan

**Keywords:** Acute kidney injury, Outpatient, Seasonality, Dehydration, Claim database, Diseases, Health care, Medical research, Nephrology, Risk factors

## Abstract

Acute kidney injury (AKI) is a clinically important condition associated with adverse outcomes. While community-acquired AKI is common, its seasonal patterns remain poorly understood, particularly in outpatient settings. This study aimed to examine the seasonality and community-acquired AKI in Japan. Of 4,401,387 individuals with at least 1 month of data in the Japanese health insurance claims database between August 2016 and July 2018, 4,282,805 were included in the analysis as outpatients. The monthly incidence of community-acquired AKI was evaluated using the incidence rate ratio (IRR) calculated by generalized estimating equations. We constructed multivariable models adjusted for sex, age, comorbidities and medications. There were 20,634 new-onset community-acquired AKI cases. The frequency of new-onset community-acquired AKI gradually increased from June to July, with the highest adjusted IRR in July (1.19 [95% confidence intervals: 1.11–1.27]). Subgroup analyses showed that IRRs for community-acquired AKI were consistently higher in July than in February across all categories. Notably, a significant interaction was observed between seasonality and age (*P* = 0.029), with the highest IRR observed in the youngest age group (< 20 years). These results suggest the potential relevance of seasonal and environmental factors in AKI prevention in outpatient settings.

**Supplementary Information:**

The online version contains supplementary material available at 10.1038/s41598-026-61190-6.

## Introduction

Acute kidney injury (AKI) is associated with a variety of negative outcomes, such as a poor prognosis, increased hospitalization rate, and higher healthcare costs^1,2^. AKI can develop in the community and hospital settings. Hospital-acquired AKI occurs in 15–20% of hospitalized patients^3^, while the rate of community-acquired AKI is greater than that occurring in the hospital setting^4^. Therefore, early detection and prevention of community-acquired AKI are important medical issues. Prerenal AKI accounts for the majority of community-acquired AKI^5^, and dehydration is known to be a major cause^6^. Dehydration reduces the amount of fluid in the body, reducing circulating plasma volume and renal blood flow, leading to the development of AKI^7–9^. Dehydration is strongly influenced by environmental factors, such as temperature and humidity, and dehydration during the summer months may affect the development of AKI. The influence of environmental factors on the development of disease has been reported for many diseases, such as asthma, migraine, and infectious diseases^10–13^, but studies on the relationship between the development of AKI and environmental factors are limited^14^.

The seasonality of AKI onset has also been reported in hospitalized patients in Japan^15^. However, compared with inpatients, who generally have appropriate temperature and fluid management, outpatients may be more susceptible to seasonal influences, including environmental factors such as temperature, because managing them appropriately is more difficult. In particular, because dehydration is more likely to occur in the summer when temperatures are higher, an association between community-acquired AKI and the season is likely. To the best of our knowledge, this possibility has not been studied in outpatients because of the difficulty in determining their background.

In this study, we used the Japanese health insurance database to investigate the seasonality of community-acquired AKI in Japan.

## Methods

### Data source

The data used in this study were obtained from the JMDC health insurance claims database (JMDC Inc., Tokyo, Japan). This database is an epidemiological claims database that has accumulated claims for inpatient, outpatient, and dispensing care from multiple health insurance associations in Japan since 2005. There are seven data tables in this database: patient (e.g., sex and date of birth), facility (e.g., number of beds and department), claim (e.g., date of admission and date of discharge), injury (e.g., injury type and outcome), drug (e.g., drug name and prescription date), medical practice (e.g., practice name and frequency), and medical supply (e.g., medical supplier’s name).

### Study design

The study included 4,401,387 individuals who had data in the JMDC claims database for at least 1 month between August 2016 and July 2018. In Japan, the majority of workers retire at the age of 65 years, and the insurance coverage changes upon retirement. A characteristic of the JMDC database is that tracking individuals beyond 65 years of age becomes difficult. Therefore, the cohort was targeted at patients younger than 65 years. A total of 4,282,805 individuals were included in the analysis, and we excluded patients who were hospitalized during the entire month.

### Definitions

The AKI event was identified from claim information according to the codes of the 10th edition of the International Statistical Classification of Diseases and Related Health Problems (ICD-10)^16^  (Supplementary Table S1). Renal anemia was excluded from the definition of AKI in this study because it was considered as a chronic kidney disease (CKD) case and may have increased the number of false-positive cases. To ensure diagnostic validity, a sensitivity analysis was performed by restricting cases to those with a confirmed diagnosis, with exclusion of suspected cases. Patients who developed AKI more than once during the study period were counted as separate events if they had different dates of medical care. Patients with a medical treatment start date before August 2016 were excluded from the analysis. We defined community-acquired AKI as cases where AKI was identified in an outpatient setting or at the time of hospital admission. Specifically, we included patients with AKI codes recorded in outpatient claims and those in hospitalization or Diagnosis Procedure Combination (DPC) claims where the AKI diagnosis date coincided with the admission date. The latter category primarily represents patients who visited the Emergency Department and were admitted on the same day, indicating that AKI had developed before hospitalization. Cases in which the AKI diagnosis was recorded after the second day of admission were defined as in-hospital AKI and were excluded from the analysis. To further evaluate the robustness of our findings for outpatient AKI, we performed an additional sensitivity analysis using strict outpatient-only inclusion criteria. In this analysis, we excluded cases diagnosed on the day of hospital admission and restricted the dataset to AKI events recorded exclusively in outpatient claims.

Comorbidities (CKD, hypertension, diabetes, and heart failure) were also defined on the basis of ICD-10 codes and AKI^14,16^  (Supplementary Table S1). Patients were considered to have comorbidities if they were treated on or after the date of the first confirmed diagnosis according to the claims.

Medications were defined according to the drug names listed in the Kyoto Encyclopedia of Genes and Genomes (KEGG) Drug database^17^, and the specific codes used for identification are listed in Supplementary Table S1. Topical medications and as-needed prescriptions were excluded from the analysis because their exact dates of use could not be determined. The duration of medication use was identified according to the prescription date and the number of days prescribed. An individual was considered to be using a medication in a given month if their treatment period overlapped with that month for at least 1 day.

We categorized the months into four seasons: spring (March–May), summer (June–September), autumn (October–November), and winter (December–February).

### Statistical analysis

We evaluated the monthly burden of AKI by including initial and recurrent AKI events. If the same individual experienced multiple AKI events within the same calendar month, they were counted only once for that specific month. Additionally, to confirm the robustness of our findings, a first-event analysis was conducted exclusively. In this specific analysis, once an individual experienced their first AKI event, they were strictly excluded from the risk set for all subsequent months. The incidence of AKI was evaluated using incidence rate ratios (IRRs) and 95% confidence intervals (CIs) calculated by generalized estimating equations (GEEs) assuming a Poisson distribution and a log link function. To account for varying observation periods, the natural logarithm of the number of observed outpatient days estimated from claims data were included as an offset term. The GEE model also incorporated a robust variance estimator to account for within-individual correlation across multiple months and years.

We constructed the three following models: a crude model; a multivariable model adjusted for sex, age, and comorbidities (CKD, hypertension, diabetes, and heart failure) as Model 1; and an additional multivariable model with further adjustment for the use of medications (non-steroidal anti-inflammatory drugs [NSAIDs], renin–angiotensin system inhibitors [RAS inhibitors], and diuretics) as Model 2. The analysis for Model 2 was conducted using a 12-month dataset from August 2017 to July 2018 to ensure an appropriate look-back period for defining medication exposure and to minimize potential misclassification. February was used as the reference month for all comparisons. To evaluate whether the association between seasonality and the incidence of AKI was modified by the patients’ characteristics, we calculated interaction *P* value by including interaction terms between the seasons and each subgroup variable (sex, age, comorbidities, and medications) in the GEE model. To account for the risk of Type I errors due to multiple comparisons, the Bonferroni correction was applied to the 11 simultaneous month-versus-February comparisons. The significance threshold was set at *P* < 0.0045. The statistical analysis was performed using JMP^®^ Pro 18.0 software (SAS Institute Inc., Cary, NC, USA) and R version 4.4.2.

This study was approved by the Ethics Committee of the Department of Epidemiology and General Medicine, Graduate School of Life Sciences, Kumamoto University (Approval No.: 2270). All methods were performed in accordance with the relevant guidelines and regulations, including the contractual requirements with the data provider. The requirement for individual informed consent was formally waived by the Ethics Committee because this study was a retrospective analysis based on fully and irreversibly anonymized data.

## Results

### Characteristics of the patients

In the analyzed cohort, 20,634 new-onset community-acquired AKI cases were identified. The characteristics of these cases are shown in Table 1. Patients who developed AKI more than once during the study period were evaluated only for the first episode of community-acquired AKI (initial AKI). Among the patients with initial AKI, the percentage of men was higher than that of women, and the percentage of patients with initial AKI tended to increase with age.

**Table 1:** Characteristics of cases with initial AKI in outpatients.

Variable	Cases with initial AKI in outpatients (*n* = 15,083)
Sex, *n* (%)	
Male	8,482 (56.2)
Female	6,601 (43.8)
Age, *n* (%)	
< 10s	1,208 (8.0)
10s	1,253 (8.3)
20s	1,330 (8.8)
30s	2,020 (13.4)
40s	3,384 (22.4)
50s	4,026 (26.7)
60–64	1,862 (12.3)
Comorbidities, *n* (%)	
Hypertension	4,326 (28.7)
Diabetes mellitus	3,026 (20.1)
CKD	2,412 (16.0)
Heart failure	1,063 (7.0)

AKI: acute kidney injury; CKD: chronic kidney disease.

### Seasonality of community-acquired AKI

The incidence of community-acquired AKI gradually increased from June to July. A similar trend was observed across all multivariable models. In Model 1, July showed the highest IRR of 1.19 (95% CIs: 1.11–1.27, *P* < 0.001) (Table 2, Supplementary Table S2). There was also a trend towards an increase in the incidence of AKI over the summer when the number of AKI events was used as one per person (patients who developed AKI more than once during the study period were evaluated only for the first occurrence of AKI on the claim) (Supplementary Table S3). The sensitivity analysis restricted to strict outpatient-only cases (Supplementary Table S4) and the analysis restricted to confirmed diagnoses (Supplementary Table S5) showed seasonal trends consistent with the primary analysis. The seasonal analysis showed that the incidence of AKI was highest in summer. The IRR for summer compared with winter was 1.16 (95% CIs: 1.12–1.20, *P* < 0.001) (Supplementary Table S6).

**Table 2:** Seasonal risk ratio for developing AKI in outpatients.

	Cases of AKI	Number of at-risk person-days	Incidence rate per 10,000 person-days	Crude Model IRR (95%CI)	Adjusted Model 1 IRR (95%CI)
January	1608	227,416,575	0.0707	0.97 (0.90–1.04)	0.97 (0.91–1.04)
February	1498	205,398,492	0.0729	(reference)	(reference)
March	1842	227,641,210	0.0809	1.11 (1.04–1.19)	1.10 (1.03–1.18)
April	1623	218,586,333	0.0742	1.02 (0.95–1.09)	1.01 (0.95–1.09)
May	1697	225,101,089	0.0754	1.03 (0.97–1.11)	1.03 (0.96–1.10)
June	1919	217,771,741	0.0881	1.21 (1.13–1.29)	1.19 (1.12–1.28)
July	1981	224,834,352	0.0881	1.21 (1.13–1.29)	1.19 (1.11–1.27)
August	1860	227,440,141	0.0818	1.12 (1.05–1.20)	1.15 (1.08–1.23)
September	1687	220,179,822	0.0766	1.05 (0.98–1.13)	1.08 (1.00–1.15)
October	1663	227,455,863	0.0731	1.00 (0.94–1.07)	1.02 (0.95–1.09)
November	1594	220,100,058	0.0724	0.99 (0.93–1.06)	1.01 (0.94–1.08)
December	1662	227,524,948	0.0730	1.00 (0.93–1.07)	1.01 (0.94–1.08)

AKI: acute kidney injury; IRR: incidence rate ratio; CI: confidence interval

**Crude model**: unadjusted model with offset for patient days

**Adjusted Model 1**: adjusted for age, sex, and comorbidities (chronic kidney disease, hypertension, heart failure, and diabetes mellitus)

### Subgroup analysis of the risk of community-acquired AKI based on the patients’ background

**Fig. 1:**
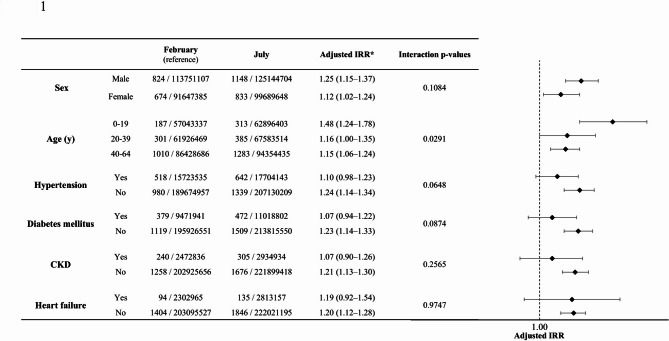
Incidence rate ratios of development of community-acquired acute kidney injury in summer (July) compared with winter (February) in patient subgroups. The values of n/N represent the number of new-onset AKI cases (n) and the total number of at-risk person-days (N) in each month. These values are provided for descriptive purposes and do not directly correspond to the estimated incidence rate ratios (IRRs). IRRs for July relative to February and their 95% confidence intervals were calculated using generalized estimating equations incorporating an offset term for observed outpatient days. All IRRs were adjusted for variables not used for stratification, including sex, age, and comorbidities (CKD, hypertension, diabetes, and heart failure), while interaction *P* values indicate the statistical significance of differences in seasonal trends between subgroups. characteristic. AKI: acute kidney injury; IRRs: incidence rate ratios; CKD: chronic kidney disease.

A subgroup analysis was performed to compare the risk of community-acquired AKI in July, which showed the peak temperature in Japan, with that in February, which showed the lowest temperature. While the point estimates of the IRR for community-acquired AKI were generally > 1.0 in most subgroups, a significant increase in July was primarily observed in certain categories, such as sex, younger age groups, and those without comorbidities (Fig. 1). Notably, a significant interaction was observed between seasonality and age (*P* interaction = 0.029), with a markedly high IRR of 1.48 (95% CIs: 1.24–1.78, *P* < 0.001) in the youngest age group (0–19 years). These findings were generally consistent in additional subgroup analyses that incorporated medication-related factors (Supplementary Fig. S1).

## Discussion

This study showed that the number of new-onset community-acquired AKI cases and IRRs gradually increased from June to July. Additionally, the subgroup analysis suggested that the point estimates for community-acquired AKI were generally highest in summer across most populations, and this trend was particularly evident in younger age groups.

Patients who developed community-acquired AKI in this study were more likely to be men, and this trend of developing AKI increased with age (Table 1). Male sex and an older age have been identified as risk factors for AKI^18,19^.

Most regions in Japan have distinct seasonal variations, with July through September being the hottest months of the year^20^. Previous studies suggested that Emergency Department visits and hospitalization due to AKI were associated with elevated ambient temperatures^14^ and that dehydration was a major cause of community-acquired AKI^6^. In this study, the risk of developing AKI gradually increased during the summer months (June–August), and the highest IRR for AKI was in July (Table 2, Supplementary Tables S4, S5). While temperature-driven dehydration is a highly plausible mechanism for this trend, notably, our findings reflect seasonal variation, and the precise underlying etiologies remain speculative. In addition to a reduction in dehydration-induced renal blood flow^9,21,22^, other summer-specific factors such as infectious gastroenteritis, rhabdomyolysis, or changes in physical activity may independently or jointly contribute to the observed increase in AKI incidence. Further studies integrating clinical records with regional meteorological data are warranted to clarify the relative contribution of these environmental and behavioral factors.

The subgroup analysis indicated that, while the point estimates for community-acquired AKI were generally higher in July than in February across most categories, a significant increase was not observed in all patient groups (Fig. 1, Supplementary Fig. S1). This finding suggests that, although a seasonal trend exists, the magnitude of the effect varies depending on the patients’ characteristics. Regarding sex, men and women showed a similar upward trend in July. The risk of developing AKI consistently increased in summer, even when stratified by age, and this risk was particularly high in young people (0–19 years). Heat stroke occurs more frequently in children in Japan in summer than in winter^23^. Severe dehydration resulting from heatstroke might be a major factor increasing the risk of community-acquired AKI in this young population. In contrast, in patients with comorbidities or those taking specific medications, the seasonal effect appeared less distinct than that in young people, with 95% CIs overlapping 1.0. This finding may be due to the relatively small number of individuals with chronic diseases or specific medication use in our study population, which consisted primarily of working-age adults. The resulting lack of statistical power in these subgroups necessitates further investigation in larger cohorts or older populations to more definitively evaluate the association between medical background and seasonality.

A strength of this study is that we showed an association between AKI and season in a non-older population, while previous reports examined the association between AKI and season in relatively older populations^15,24–27^. Furthermore, to the best of our knowledge, this is the first study to examine the effect of summer on the development of AKI by sex, age, and comorbidities.

This study has some limitations. First, this was an observational study. Therefore, there might have been unknown confounding factors that were not adjusted for. However, confounding factors that were likely to be influenced by seasonality in this study (e.g., hypertension, heart failure, and medication use) were considered. Consequently, we believe that other confounding factors would not have altered our results on seasonality. Second, the occurrence of AKI and comorbidities was identified by the disease name on the claims using ICD-10 codes. The data used in this study did not include laboratory data. Therefore, we were not able to identify the disease by laboratory values or by following fluctuations in these values. While previous research reported a low sensitivity (60%) but high specificity (86%) for identifying AKI by ICD-10 codes^28^, this may have led to an underestimation of its true incidence. Furthermore, we cannot exclude the possibility that diagnostic sensitivity varies by season. An example of this sensitivity is that increased clinical attention to dehydration or heat-related illnesses during summer might lead to more frequent coding of AKI by physicians, potentially introducing a seasonal reporting bias. We were unable to directly assess the seasonal stability of coding accuracy because of the lack of laboratory data. Therefore, our findings should be interpreted with caution. Future validation studies integrating laboratory-based clinical records are warranted to determine whether seasonal variations in coding behavior significantly affect the observed trends. Third, the JMDC claims database used in this study does not include individuals aged 75 years or older. Additionally, although the database contains some data for those aged 65–74 years, this population is underrepresented compared with the general Japanese population because of retirement and insurance transitions. Therefore, we excluded individuals aged 65 years and older from the analysis to ensure analytical stability. Consequently, our findings regarding the seasonality of AKI are specifically applicable to the working-age population younger than 65 years. Although seasonal patterns were observed even in this relatively young cohort, these results cannot be directly extrapolated to older people who are at higher risk for dehydration. Further research using databases that include older populations is necessary to confirm the effect of seasonality across all age groups. Furthermore, the JMDC database primarily comprises insured employees and their dependents, which may have introduced a “healthy worker effect.” Therefore, our study population likely represents a relatively healthier segment of the general population, excluding individuals not captured in this database who may have different risk profiles for AKI. Additionally, we could not account for occupational heat exposure, which could act as a confounder for those working in high-temperature environments. These factors should be considered when generalizing our findings to the broader population. Fourth, we might not have been able to accurately determine true community-acquired AKI. In this study, community-acquired AKI was defined as the development of AKI in patients whose claim type was outside the hospital. If the claim type was hospitalization, but AKI was the reason for hospitalization, the onset of AKI was likely to have been outside the hospital. However, completely distinguishing between these cases and AKI that developed during hospitalization is difficult. As a result, the number of community-acquired AKI cases may have been even greater than that estimated in this study. However, the purpose of this study was not to calculate the true number of patients with community-acquired AKI, but to examine the seasonality of AKI development in outpatients. Only AKI cases that were more reliably considered outpatients were considered. To address this potential diagnostic bias, we performed a sensitivity analysis restricted to strict outpatient-only cases (Supplementary Table S4) and an analysis restricted to confirmed diagnoses (Supplementary Table S5), which showed seasonal trends consistent with the primary analysis. Fifth, because this database does not include the locations where patients developed AKI, we were unable to perform a regional sensitivity analysis. Japan spans a wide climatic range from north to south, and the effect of temperature on AKI may vary by region. Although there is a consistent nationwide trend of high temperatures in summer and low temperatures in winter, our inability to account for regional climatic differences is a limitation. Future studies using databases with geographic information are necessary to clarify how regional climate variations affect the seasonality of AKI. Sixth, our study period was limited to only 2 years. Although the summer of 2018 was hotter than average^29^, consistent seasonal increases were observed in 2017 and 2018 (Supplementary Table S3). Nevertheless, further validation over longer periods is necessary to confirm the generalizability of these patterns. Seventh, the use of medications was identified solely on the basis of medical claims data, which presents some limitations. Information regarding over-the-counter medications was unavailable in the database. Additionally, topical medications were excluded from the analysis because their exact dates of use could not be accurately identified from the claim records. While we adjusted for major medications known to affect kidney function, the possibility of residual confounding by other drugs cannot be ruled out. Therefore, further studies are required to more comprehensively evaluate the effect of a wider range of medications on the seasonal variation of AKI.

In conclusion, this study showed that the incidence of community-acquired AKI gradually increased during the summer among working-age adults younger than 65 years in Japan. These findings highlight the potential relevance of seasonal and environmental factors in AKI prevention in outpatient settings. However, the present claims-based analysis evaluated calendar-based seasonality and did not directly assess meteorological or regional exposure data, such as temperature, humidity, heat index, individual heat exposure, or regional climatic variation. Therefore, future studies incorporating laboratory data, clinical information, and meteorological and regional exposure data are needed to clarify the role of climatic and other environmental factors in AKI incidence. Such evidence may help inform seasonally tailored strategies for AKI prevention in outpatient settings.

## Supplementary Information

Below is the link to the electronic supplementary material.

Supplementary Material 1

Supplementary Material 2

## Data Availability

We utilized a commercial database (e.g., “JMDC Claims Database”) for our study. However, due to licensing restrictions and the proprietary nature of the data, we are unable to publicly share the dataset used in this research. Interested parties may obtain access to the dataset directly from the database provider, subject to their terms and conditions.
