# Supplementary material for: Seasonality of acute kidney injury incidence in Japanese outpatients
Source: Sci Rep. 2026 Jul 27;16:22259. doi: 10.1038/s41598-026-61190-6 (PMC13407886; doi:10.1038/s41598-026-61190-6)
Supplement: Supplementary file 1 — Supplementary Material 1 [file 41598_2026_61190_MOESM1_ESM.docx]

**Seasonality of acute kidney injury incidence in Japanese outpatients**

Yuka Sakazaki^1¶^, Yuki Kondo*^1¶^, Mizuki Okuma^1^, Ayaka Seki^1^, Takamasa Sakai^2^, Tetsumi Irie^3^, Yoichi Ishitsuka^1^

^1^Department of Clinical Chemistry and Informatics, Graduate School of Pharmaceutical Sciences, Kumamoto University, 5-1 Oehommachi Chuo-ku, Kumamoto, Japan

^2^Drug Informatics, Faculty of Pharmacy, Meijo University, 150 Yagotoyama, Tempaku-ku, Nagoya, Japan

^3^Department of Pharmaceutical Packaging Technology, Faculty of Life Sciences, Kumamoto University, 5-1 Oehommachi Chuo-ku, Kumamoto, Japan

*Corresponding author

Yuki Kondo, Ph.D.

5-1 Oehommachi Chuo-ku, Kumamoto 862-0973, Japan

E-mail: ykondo@kumamoto-u.ac.jp

Tel: +81-96-371-4559

^¶^These authors equally contributed to this work.

**Supplemental Material**

**Supplementary Table S1.** Common diagnoses in each admission diagnosis category of patients.

**Supplementary** **Table S2.** Incidence rate ratios of AKI in outpatients adjusted for medication use.

**Supplementary Table S3.** Monthly incidence of total and first-episode acute kidney injury in an insured population.

**Supplementary** **Table S4.** Incidence rate ratios of AKI according to seasonality in the sensitivity analysis restricted to strict outpatient-only cases.

**Supplementary Table S5.** Incidence rate ratios of AKI according to seasonality in the sensitivity analysis restricted to confirmed diagnoses (excluding suspected cases).

**Supplementary Table S6.** Comparison of AKI incidence rate ratios across the four seasons.

| **Supplementary Table S1.** Common diagnoses in each admission diagnosis category and the definition of drug groups based on ICD-10 and KEGG DGROUP codes. | |
| --- | --- |
| **Diagnosis category** | **ICD-10 code** |
| Acute kidney injury | N14, N17, N19, R34* |
| Chronic kidney disease | I12, I13, N01, N03, N04, N05, N06, N07, N08, N14, N15, N18, N19, N25, N26, N13.7, N28.0, N28.8, N39.1 |
| Hypertension | I10–I15 |
| Diabetes mellitus | E10–E14 |
| Heart failure | I25.5, I50.0, I50.1, I50.9, J81 |
| **Drug Group** | **KEGG DGROUP** |
| Non-steroidal anti-inflammatory drugs | DG1504 |
| Renin-angiotensin system inhibitors | DG01925 |
| Diuretics | DG01690, DG01746, DG01747, DG01748,  DG01749, DG01885, DG02992 |
| ICD-10: 10th edition of the International Statistical Classification of Diseases and Related Health Problems.  KEGG: Kyoto Encyclopedia of Genes and Genomes  *Renal anemia was excluded from the definition of AKI in this study because it was considered as a chronic kidney disease case and may have increased the number of false-positive cases. | |

| **Supplementary Table S2.** Incidence rate ratios of AKI in outpatients adjusted for medication use. | | | | | | |
| --- | --- | --- | --- | --- | --- | --- |
|  | **Cases of AKI** | **Number of at-risk person-days** | **Incidence rate**  **per 10,000 person-days** | **Crude Model**  **IRR (95%CI)** | **Adjusted Model 1**  **IRR (95%CI)** | **Adjusted Model 2**  **IRR (95%CI)** |
| **January** | 831 | 113352779 | 0.0733 | 0.98 (0.89–1.08) | 0.99 (0.90–1.09) | 0.98 (0.89–1.08) |
| **February** | 764 | 102366688 | 0.0746 | (reference) | (reference) | (reference) |
| **March** | 920 | 113455794 | 0.0811 | 1.09 (0.99–1.19) | 1.08 (0.99–1.19) | 1.08 (0.99–1.19) |
| **April** | 797 | 107955161 | 0.0738 | 0.99 (0.90–1.09) | 0.99 (0.90–1.09) | 0.99 (0.90–1.09) |
| **May** | 859 | 111161979 | 0.0773 | 1.04 (0.94–1.14) | 1.03 (0.94–1.13) | 1.03 (0.94–1.14) |
| **June** | 944 | 107539650 | 0.0878 | 1.18 (1.07–1.29) | 1.16 (1.06–1.28) | 1.17 (1.07–1.28) |
| **July** | 953 | 111030357 | 0.0858 | 1.15 (1.05–1.26) | 1.14 (1.04–1.25) | 1.14 (1.04–1.25) |
| **August** | 963 | 113477462 | 0.0849 | 1.14 (1.04–1.25) | 1.17 (1.06–1.28) | 1.17 (1.07–1.28) |
| **September** | 874 | 109813541 | 0.0796 | 1.07 (0.97–1.17) | 1.09 (0.99–1.20) | 1.09 (0.99–1.20) |
| **October** | 845 | 113430306 | 0.0745 | 1.00 (0.91–1.10) | 1.01 (0.92–1.11) | 1.01 (0.92–1.11) |
| **November** | 742 | 109743841 | 0.0676 | 0.91 (0.82–1.00) | 0.92 (0.83–1.01) | 0.91 (0.83–1.01) |
| **December** | 827 | 113444456 | 0.0729 | 0.98 (0.89–1.07) | 0.98 (0.89–1.08) | 0.98 (0.89–1.08) |
| AKI: acute kidney injury; IRR: incidence rate ratio; CI: confidence interval.  **Crude model:** unadjusted model with offset for patient days.  **Adjusted Model 1:** adjusted for age, sex, and comorbidities (chronic kidney disease, hypertension, heart failure, and diabetes mellitus).  **Adjusted Model 2:** adjusted for age, sex, comorbidities (chronic kidney disease, hypertension, heart failure, diabetes mellitus), and medications (non-steroidal anti-inflammatory drugs, renin-angiotensin system inhibitors, and diuretics). **Note:** This analysis was conducted using a 12-month dataset (August 2017 to July 2018) to ensure an appropriate look-back period for defining medication exposure and to minimize potential misclassification. | | | | | | |

| Supplementary Table S3. Monthly Incidence of Total and First-Episode Acute Kidney Injury in an Insured Population. | | |
| --- | --- | --- |
|  | **Total AKI Episodes, *n* (Incidence rate per 10,000 person-days)** | **Initial AKI Cases, *n***  **(Incidence rate per 10,000 person-days)** |
| Aug 2016 | 897 (0.079) | 897 (0.079) |
| Sep 2016 | 813 (0.074) | 774 (0.070) |
| Oct 2016 | 818 (0.072) | 745 (0.065) |
| Nov 2016 | 852 (0.077) | 751 (0.068) |
| Dec 2016 | 835 (0.073) | 715 (0.063) |
| Jan 2017 | 777 (0.068) | 601 (0.053) |
| Feb 2017 | 734 (0.071) | 561 (0.055) |
| Mar 2017 | 922 (0.081) | 676 (0.059) |
| Apr 2017 | 826 (0.075) | 630 (0.057) |
| May 2017 | 838 (0.074) | 608 (0.053) |
| Jun 2017 | 975 (0.088) | 714 (0.065) |
| Jul 2017 | 1028 (0.090) | 737 (0.065) |
| Aug 2017 | 963 (0.085) | 653 (0.058) |
| Sep 2017 | 874 (0.080) | 595 (0.054) |
| Oct 2017 | 845 (0.074) | 564 (0.050) |
| Nov 2017 | 742 (0.068) | 485 (0.044) |
| Dec 2017 | 827 (0.073) | 527 (0.047) |
| Jan 2018 | 831 (0.073) | 536 (0.047) |
| Feb 2018 | 764 (0.075) | 475 (0.047) |
| Mar 2018 | 920 (0.081) | 540 (0.048) |
| Apr 2018 | 797 (0.074) | 508 (0.047) |
| May 2018 | 859 (0.077) | 562 (0.051) |
| Jun 2018 | 944 (0.088) | 617 (0.058) |
| Jul 2018 | 953 (0.086) | 612 (0.055) |

| **Supplementary Table S4.** Incidence rate ratios of AKI according to seasonality in the sensitivity analysis restricted to strict outpatient-only cases. | | | | | |
| --- | --- | --- | --- | --- | --- |
|  | **Cases of AKI** | **Number of at-risk person-days** | **Incidence rate**  **per 10,000 person-days** | **Crude Model**  **IRR (95%CI)** | **Adjusted Model 1**  **IRR (95%CI)** |
| **January** | 1548 | 227416575 | 0.0681 | 0.97 (0.90–1.04) | 0.97 (0.90–1.04) |
| **February** | 1445 | 205398492 | 0.0704 | (reference) | (reference) |
| **March** | 1790 | 227641210 | 0.0786 | 1.12 (1.04–1.20) | 1.11 (1.04–1.19) |
| **April** | 1561 | 218586333 | 0.0714 | 1.02 (0.95–1.09) | 1.01 (0.94–1.09) |
| **May** | 1632 | 225101089 | 0.0725 | 1.03 (0.96–1.10) | 1.02 (0.96–1.10) |
| **June** | 1848 | 217771741 | 0.0849 | 1.21 (1.13–1.29) | 1.19 (1.12–1.28) |
| **July** | 1910 | 224834352 | 0.0850 | 1.21 (1.13–1.29) | 1.19 (1.11–1.27) |
| **August** | 1770 | 227440141 | 0.0778 | 1.11 (1.03–1.18) | 1.14 (1.06–1.22) |
| **September** | 1617 | 220179822 | 0.0734 | 1.04 (0.97–1.12) | 1.07 (1.00–1.15) |
| **October** | 1605 | 227455863 | 0.0706 | 1.00 (0.94–1.08) | 1.02 (0.95–1.09) |
| **November** | 1524 | 220100058 | 0.0692 | 0.98 (0.92–1.06) | 1.00 (0.93–1.07) |
| **December** | 1589 | 227524948 | 0.0698 | 0.99 (0.92–1.07) | 1.00 (0.93–1.07) |
| AKI: acute kidney injury; IRR: incidence rate ratio; CI: confidence interval.  **Crude model:** unadjusted model with offset for patient days.  **Adjusted Model 1:** adjusted for age, sex, and comorbidities (chronic kidney disease, hypertension, heart failure, and diabetes mellitus). | | | | | |

| **Supplementary Table S5.** Incidence rate ratios of AKI according to seasonality in the sensitivity analysis restricted to confirmed diagnoses (excluding suspected cases)**.** | | | | | |
| --- | --- | --- | --- | --- | --- |
|  | **Cases of AKI** | **Number of at-risk person-days** | **Incidence rate**  **per 10,000 person-days** | **Crude Model**  **IRR (95%CI)** | **Adjusted Model 1**  **IRR (95%CI)** |
| **January** | 252 | 227416575 | 0.0111 | 1.08 (0.90–1.29) | 1.09 (0.91–1.31) |
| **February** | 211 | 205398492 | 0.0103 | (reference) | (reference) |
| **March** | 250 | 227641210 | 0.0110 | 1.07 (0.89–1.28) | 1.06 (0.88–1.26) |
| **April** | 273 | 218586333 | 0.0125 | 1.22 (1.02–1.45) | 1.20 (1.00–1.43) |
| **May** | 267 | 225101089 | 0.0119 | 1.15 (0.97–1.38) | 1.12 (0.94–1.35) |
| **June** | 286 | 217771741 | 0.0131 | 1.28 (1.07–1.52) | 1.23 (1.03–1.46) |
| **July** | 309 | 224834352 | 0.0137 | 1.34 (1.12–1.59) | 1.27 (1.07–1.51) |
| **August** | 356 | 227440141 | 0.0157 | 1.52 (1.29–1.80) | 1.65 (1.39–1.94) |
| **September** | 285 | 220179822 | 0.0129 | 1.26 (1.06–1.50) | 1.34 (1.12–1.60) |
| **October** | 247 | 227455863 | 0.0109 | 1.06 (0.88–1.27) | 1.11 (0.92–1.33) |
| **November** | 274 | 220100058 | 0.0124 | 1.21 (1.01–1.45) | 1.26 (1.05–1.50) |
| **December** | 265 | 227524948 | 0.0116 | 1.13 (0.95–1.36) | 1.16 (0.97–1.39) |
| AKI: acute kidney injury; IRR: incidence rate ratio; CI: confidence interval.  **Crude model:** unadjusted model with offset for patient days.  **Adjusted Model 1:** adjusted for age, sex, and comorbidities (chronic kidney disease, hypertension, heart failure, and diabetes mellitus). | | | | | |

| **Supplementary Table S6.** Comparison of AKI incidence rate ratios across the four seasons. | | | | | |
| --- | --- | --- | --- | --- | --- |
| **Season** | **Mean monthly AKI cases** | **Number of at-risk person-days** | **Incidence rate**  **per 10,000 person-days** | **Crude Model**  **IRR (95%CI)** | **Adjusted Model 1**  **IRR (95%CI)** |
| **Spring** | 1721 | 671328632 | 0.0769 | 1.06 (1.03–1.11) | 1.06 (1.02–1.10) |
| **Summer** | 1862 | 890226056 | 0.0837 | 1.16 (1.12–1.20) | 1.16 (1.12–1.20) |
| **Autumn** | 1629 | 447555921 | 0.0728 | 1.01 (0.97–1.05) | 1.02 (0.98–1.07) |
| **Winter** | 1589 | 660340015 | 0.0722 | (reference) | (reference) |
| AKI: acute kidney injury; IRR: incidence rate ratio; CI: confidence interval. **Seasons:** spring (March–May), summer (June–September), autumn (October–November), and winter (December–February).  **Crude model:** unadjusted model with offset for patient days.  **Adjusted Model 1:** adjusted for age, sex, and comorbidities (chronic kidney disease, hypertension, heart failure, and diabetes mellitus). | | | | | |
