# Supplementary material for: Seasonality of acute kidney injury incidence in Japanese outpatients
Source: Sci Rep. 2026 Jul 27;16:22259. doi: 10.1038/s41598-026-61190-6 (PMC13407886; doi:10.1038/s41598-026-61190-6)
Supplement: Supplementary file 2 — Supplementary Material 2 [file 41598_2026_61190_MOESM2_ESM.docx]

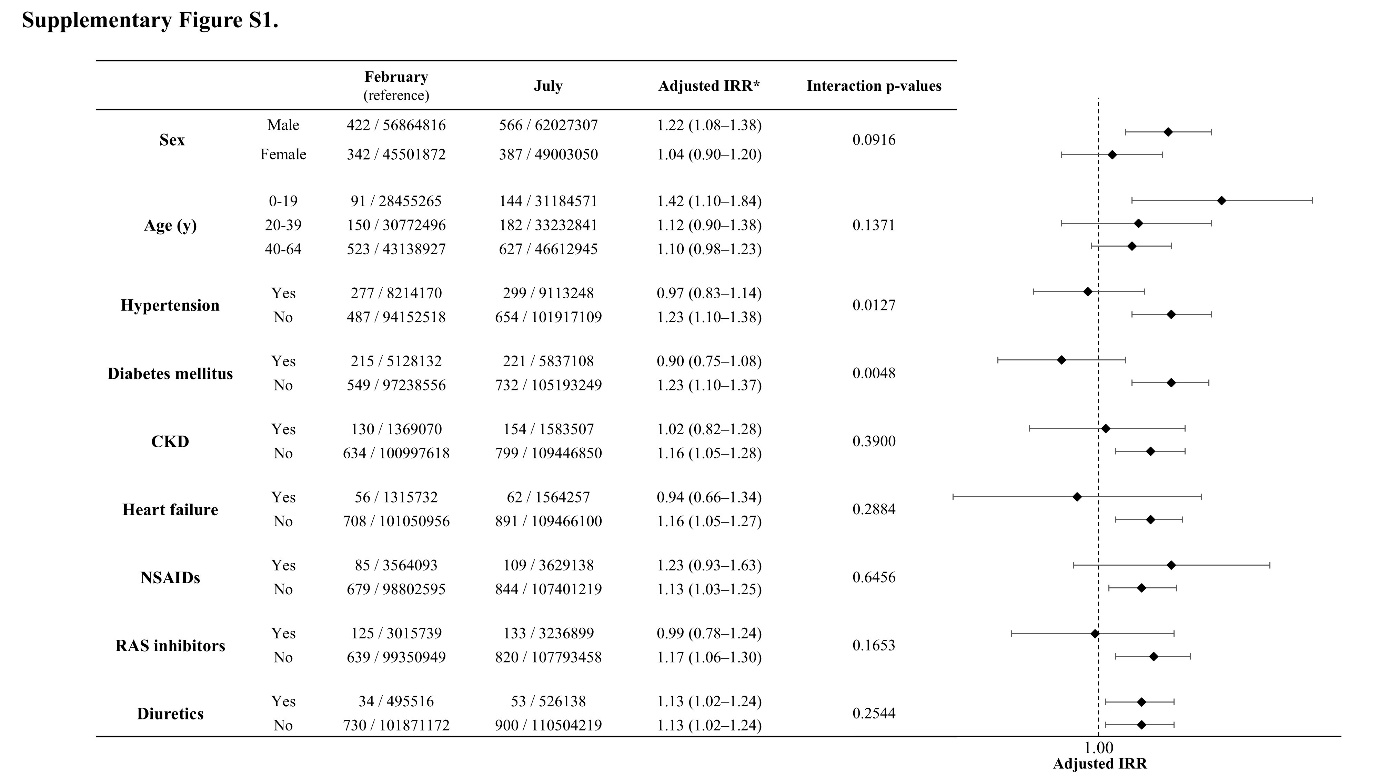


Subgroup analysis of incidence rate ratios for community-acquired AKI in July compared with February, with adjustment for medication use. The values of n/N represent the number of new-onset AKI cases (n) and the total number of at-risk person-days (N) in each month. These values are provided for descriptive purposes and do not directly correspond to the estimated incidence rate ratios (IRRs). IRRs for July relative to February and their 95% confidence intervals were estimated using generalized estimating equations with a Poisson distribution, based on a 12-month dataset from August 2017 to July 2018 to ensure an appropriate look-back period for defining medication exposure and to minimize potential misclassification. All IRRs were adjusted for factors not used for stratification, including sex, age, comorbidities (chronic kidney disease, hypertension, diabetes, and heart failure), and medications (non-steroidal anti-inflammatory drugs, renin–angiotensin system inhibitors, and diuretics), with interaction *P* values provided to evaluate the modification of seasonal effects by each characteristic.

AKI: acute kidney injury; IRRs: incidence rate ratios; CKD: chronic kidney disease; NSAIDs: non-steroidal anti-inflammatory drugs; RAS inhibitors: renin–angiotensin system inhibitors.
